# Total arch replacement for aortic arch aneurysm with coexisting middle aortic syndrome

**DOI:** 10.1016/j.ijscr.2018.11.049

**Published:** 2018-11-24

**Authors:** Zaiqiang Yu, Masahito Minakawa, Norihiro Kondo, Kazuyuki Daitoku, Ikuo Fukuda

**Affiliations:** Department of Thoracic and Cardiovascular Surgery, Hirosaki University Graduate School of Medicine, Aomori, Japan

**Keywords:** MAS, middle aortic syndrome, TAA, thoracic aortic aneurysm, ARSCA, aberrant right subclavian artery, CPB, cardiopulmonary bypass, SCP, selective cerebral perfusion, Aortic arch aneurysm, Middle aortic syndrome, Axillary-femoral artery bypass, Case report

## Abstract

Introduction: Middle aortic syndrome (MAS) combined with thoracic aortic aneurysm (TAA) is a rare vascular disease. One stage open surgery to treat this condition, becomes a challenge for our cardiovascular surgery.

Presentation of case: A 69-year-old man presented with a saccular type aortic arch aneurysm, shaggy aorta and severe atherosclerotic stenosis of the thoracoabdominal aorta with middle aortic syndrome and aberrant right subclavian artery, renovascular hypertension, renal dysfunction, and intermittent claudication of both legs. Total arch replacement procedure was performed under a cardiopulmonary bypass using aortic inflow from the right axillary artery and a femoro-femoral crossover bypass graft to avoid malperfusion of the lower body. Before weaning from the cardiopulmonary bypass, we established an extra-anatomical bypass from the ascending aortic graft to the femoro-femoral crossover bypass graft. 3D-CT showed patency of bypass graft without any sign of stenosis postoperative. The patient’s postoperative course was uneventful and he was discharged from hospital with improvements in intermittent claudication, hypertension, and renal dysfunction.

Discussion: Although open surgery including graft bypass for MAS is more invasive than endovascular treatment, it could be performed successfully to preventing from intraoperative complication or complications postoperatively.

Conclusion: Combined operation of total arch replacement and a bypass from the ascending aorta to the bifemoral arteries is alternative for MAS combined with TAA.

## Highlights

• Middle aortic syndrome (MAS) combined with thoracic aortic aneurysm (TAA) is a rare vascular disease.
• Combined operation of total arch replacement and a bypass from the ascending aorta to the bifemoral arteries is alternative for MAS combined with TAA.
• One stage open surgery got good clinical outcome for this condition without any another intervention postoperative.

## 1 Introduction

Middle aortic syndrome (MAS) is a rare vascular disease that presents as severe stenosis of the middle of the thoracoabdominal aorta. The incidence ranges from 0.5% to 2% of the population [1]. Catheter intervention and endovascular surgery are minimally invasive approaches to treating MAS. Open aortic surgery using a cardiopulmonary bypass is an alternative to endovascular procedures, but it can result in malperfusion of the abdominal organs and legs. We describe total aortic arch replacement to treat coexisting MAS under a cardiopulmonary bypass with dual aortic inflow through the axillary artery and a femoro-femoral bypass graft. Our work has been reported in line with the SCARE criteria [2].

## 2 Presentation of case

**Fig. 1:**
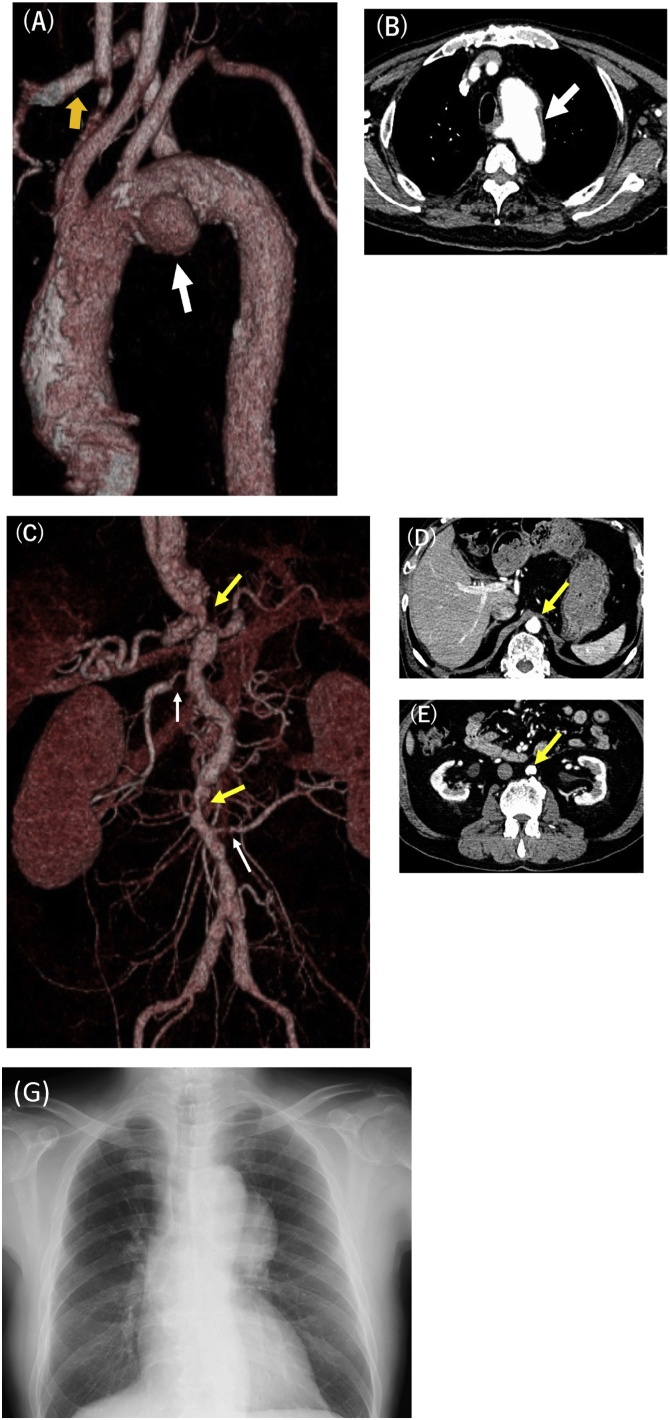
Preoperative computed tomography images. (A) Saccular type aortic arch aneurysm (solid arrow) and aberrant right subclavian artery (white arrow). (B) Shaggy aorta was acknowledged (white arrow). (C) Aortic stenosis at the level of the thoracoabdominal aorta and infra-renal abdominal aorta (gold arrows). Right renal artery had severe stenosis (white arrow). (D, E) Abdominal aorta was 19 mm just above celiac artery (CA), it become more stenosis at renal level (13 mm).

A 69-year-old man presented with severe intermittent claudication, and chronic kidney disease (Serum Creatinine 1.7 mg/dL, eGFR 32.2 mL/min/1.73 m^2^), was introduced to our department. Renovascular hypertension induced by renal arteries stenosis had become exacerbated despite an increased dose of oral anti-hypertensive drugs (Telmisartan 40 mg, Doxazosin mesylate 4 mg, Amlodipine besilate 7.5 mg). He also had a history of autoimmune pancreatitis and diabetes mellitus. Electrocardiogram was normal without any arrhythmia. Contrast-enhanced computerized tomography (CT) revealed a saccular aortic aneurysm with a diameter of 68 mm on the middle aortic arch (Fig. 1A) without any relevant complications. An aberrant right subclavian artery (ARSCA) originated from the distal aortic arch, and a thick atheroma irregularly protruded from the intima of the aortic arch and descending thoracic aorta. Severe diffuse aortic stenosis (Fig. 1C, D, E) was confirmed in the thoracoabdominal and infra-renal abdominal aortae with severe calcification and shaggy aorta (Fig. 1B). The orifice of the bilateral renal arteries was also stenosed and calcified (Fig. 1C). Chest X-ray showed enlargement of aortic arch (Fig. 1G). Pulses were not palpable on the bilateral common femoral arteries. Therefore, surgical intervention was indicated for both the aortic arch aneurysm with ARSCA and MAS. The patient underwent total aortic arch replacement and a concomitant extra-anatomical bypass of the bilateral femoral arteries.

**Fig. 2:**
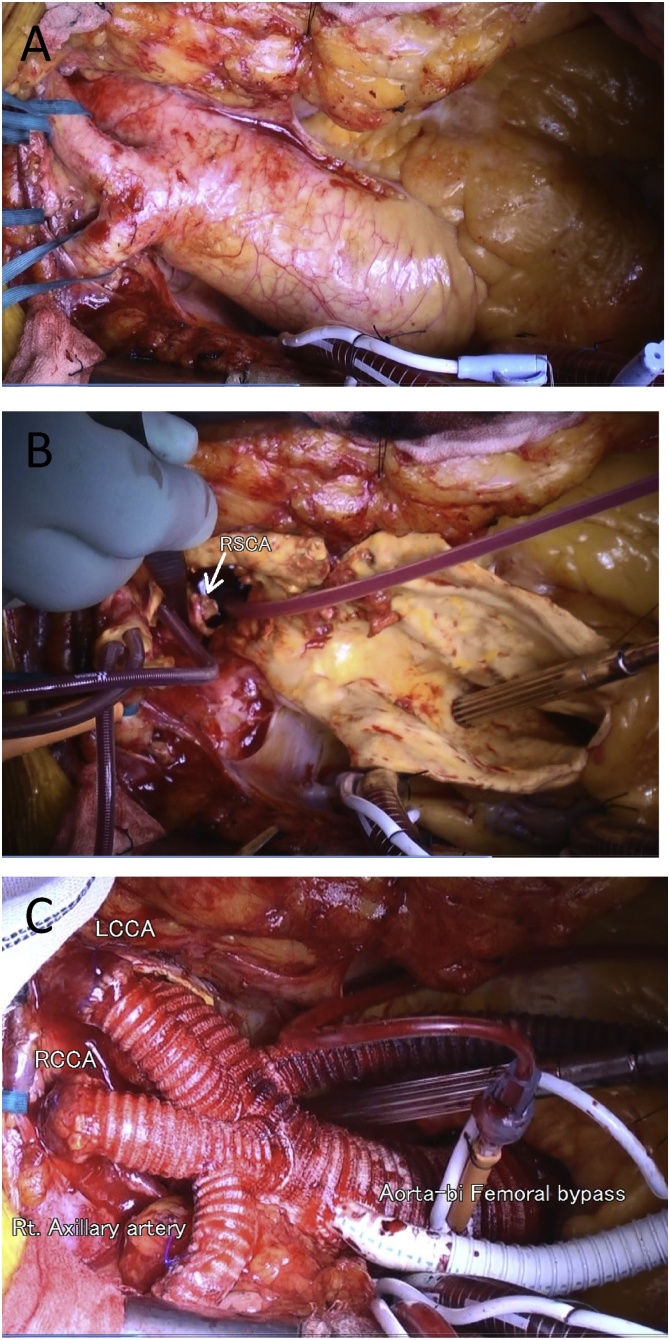
Postoperative three-dimensional computed tomography findings. Graft of total arch replacement (a) at the area from the sinotubular junction (unfilled arrow) to the distal aortic arch (filled arrow). Extra-anatomical bypass is established to the right axillary artery (b) and graft from total arch replacement to the femoro-femoral crossover bypass graft (c). LAx, left axillary artery; LCCA, left common carotid artery; RAx, right axillary artery; RCCA, right common carotid artery.

After preparing a median sternotomy and before establishing a cardiopulmonary bypass (CPB), we created a femoro-femoral crossover bypass using a T-shaped, 8-mm ringed, extended polytetrafluoroethylene (ePTFE) graft. A 9-mm Dacron graft was anastomosed to the right axillary artery and CPB perfusion proceeded via a femora-femoral crossover bypass graft for the lower extremities and a right axillary artery graft for the upper extremities. Under deep hypothermic circulatory arrest at a bladder temperature of 25 °C, the aortic arch was transected at the distal portion of the orifice of the aberrant right subclavian artery (Fig. 2A, B). Selective cerebral perfusion (SCP) was established including right and left common carotid artery and left subclavian artery. Right subclavian artery was perfused from inflow graft after the stump of ARSCA was sutured and closed at proximal site from aortic arch. A 26-mm SHIELD NEO ^™^ graft (Japan Lifeline Co. Ltd., Tokyo, Japan) was used for total aortic replacement to treat the aortic aneurysm. Thereafter, the arch vessels were reconstructed. Graft bypass was performed from aortic graft branch for reconstructing right subclavian artery in the anatomic position. During systemic rewarming, inflow anastomoses of extra-anatomic bypass graft from the ascending aortic graft to the femoro-femoral crossover bypass graft was established to maintain circulation blood perfusion to the lower body for MAS (Fig. 2C). The total durations of CPB, cardiac arrest, and circulatory arrest were 252, 137 and 69 min, respectively.

**Fig. 3:**
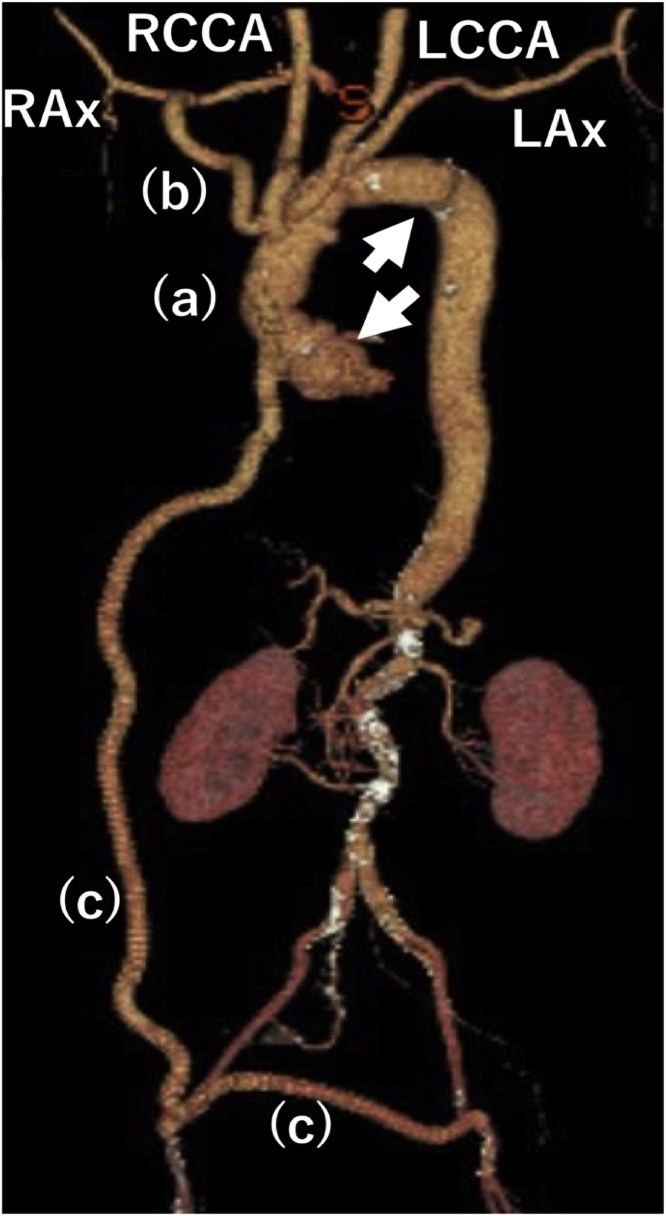
Intro-operative picture. A: aorta. B: aberrant right subclavian artery. C: Total image of total procedure finished.

**Fig. 4:**
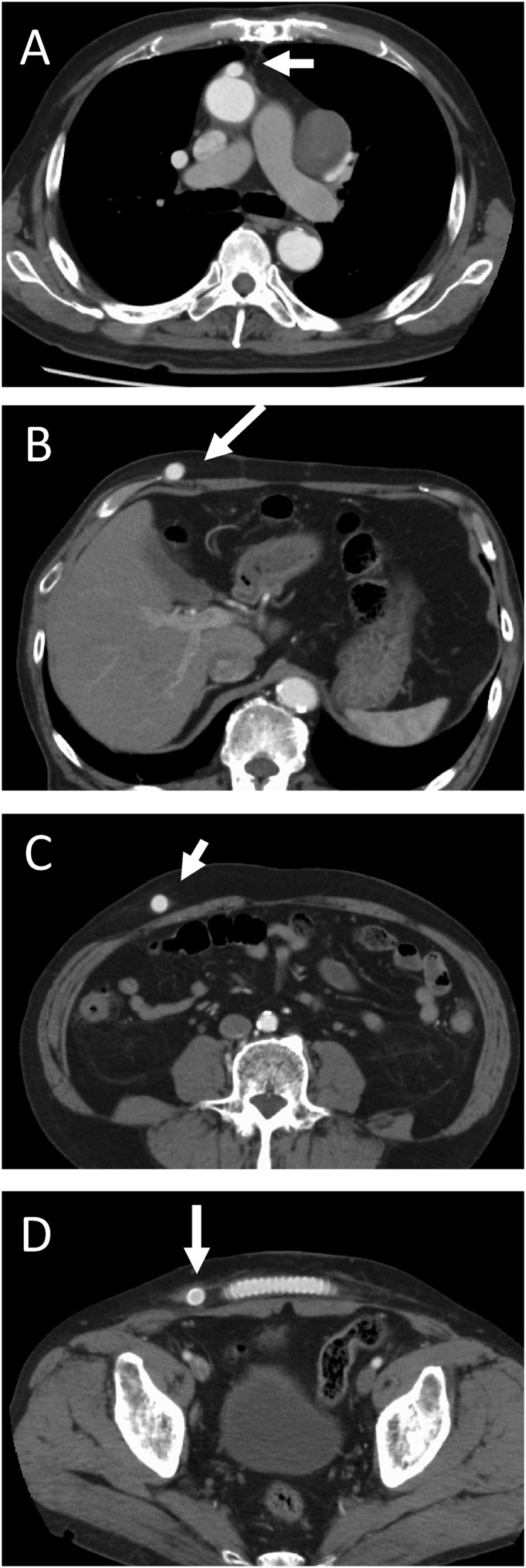
Computed tomography findings showed that graft was patency (white arrow).

The postoperative course was uneventful and the patient was discharged without complications. The serum creatinine level was postoperatively improved (Cre 1.1 mg/dL). Blood pressure was normalized with lower doses of anti-hypertensive drugs (Amlodipine besilate 5 mg), the ankle brachial pressure index was almost normal (right: 0.92, Left: 0.86), and the intermittent claudication disappeared. Three-dimensional CT showed that all grafts were patent without stenosis (Fig. 3). Pathological diagnosis showed that atheroma change was found mainly. Edoxaban tosilate hydrate 30 mg/day was prescribed for keep graft patency. There was no any complications induced by graft stenosis or occlusion two years postoperative, the bypass graft to femoral arteries is patency (Fig. 4).

## 3 Discussion

Middle aortic stenosis (MAS) is a rare aortic disease that can be congenital or adult-onset. The typical etiology of MAS is congenital coarctation, aortitis, and atherosclerosis of the aorta. The renal artery is involved in 50–90% of patients with MAS [3,4]. Many symptoms and comorbidities are associated with MAS due to reduced blood circulation to the visceral organs and lower extremities; they include abdominal angina, renovascular hypertension, chronic kidney disease, and intermittent claudication [3,5]. Comorbid MAS and a thoracic aortic aneurysm is rare [6]. Middle aortic syndrome in adults was originally described as a variant of Takayasu aortitis secondary to localized aortitis [7]. However, our patient did not have a history of fever or an elevated inflammatory response. Atherosclerosis might have been the most probable cause, because intimal thickening, severe calcification, and shaggy atherosclerotic changes were found on the thoracoabdominal aortic segment.

An extra-anatomical bypass is an effective surgical strategy for managing MAS. The ascending aorta or the descending thoracic aorta can serve as an inflow site for a bypass to the lower abdominal aorta or iliac artery [8]. Hetzer found that an extra-anatomic bypass resulted in good outcomes and provided long-term graft patency in children with MAS [9]. Resection and interposition grafts at stenotic sites represent another option [10].

Percutaneous intervention is also an effective alternative to bypass surgery, but it is associated with a high incidence of restenosis and reintervention [11]. Our patient had diffuse stenosis of the thoracoabdominal aorta with severe calcification over the orifice of the renal artery. Regarding endovascular repair for this case, it is difficult to get enough landing zone, and with high risk of aortic rupture or restenosis. Although endovascular repair is as the first line in management for these patients as well for MAS, but in the presence of peripheral vascular disease, this would not be easily feasible. We decided to implement a bypass from the ascending aorta to the bilateral-femoral arteries using inflow from the graft for the total arch replacement as CT images. This simple procedure allows secure perfusion of the lower extremities during CPB. Renovascular hypertension was also improved after the surgery without the need for secondary intervention to the renal arteries.

This case highlights that thoracic aortic aneurysm with concomitant MAS can be treated by combined operation of total arch replacement and a bypass from the ascending aorta to the bifemoral arteries successfully. Visceral and legs malperfusion was prevented by femoral arteries perfusion, and MAS was treated by graft bypass rather than graft replacement of stenosis section which is easy to be performed. The clinical outcome was satisfactory.

## Conflicts of interest

There are no any conflicts of interest to disclose.

## Funding

There are no any sources of funding for this study to declare.

## Ethical approval

This study had got ethical approval from the institutional review boards of the Hospital of Hirosaki University.

## Consent

Written, informed consent was obtained from the patient for the publication of this case report and any accompanying images.

## Author contribution

Zaiqiang Yu, Masahito Minakawa and Ikuo fukuda performed the surgery.

All authors drafted the manuscript.

All authors read and approved the final manuscript.

## Registration of research studies

Researchregistry4411.

## Guarantor

Masahito Minakawa.

Department of Thoracic and Cardiovascular Surgery, Hirosaki University Graduate

School of Medicine, 5 Zaifu-cho, Hirosaki, Aomori 036-8562, Japan.

## Provenance and peer review

Not commissioned, externally peer reviewed.
